# Superior mesenteric artery outcomes after large fenestration strut relocation with the Zenith Fenestrated endoprosthesis

**DOI:** 10.1186/s42155-020-00148-9

**Published:** 2020-10-25

**Authors:** Aleem K. Mirza, Timothy M. Sullivan, Nedaa Skeik, Jesse Manunga

**Affiliations:** Section of Vascular and Endovascular Surgery, Minneapolis Heart Institute, Abbott Northwestern Hospital, 920 E. 28th Street, Suite 200, Minneapolis, MN 55407 USA

**Keywords:** Superior mesenteric artery (SMA), Zenith Fenestrated AAA Endovascular® (ZFen), Physician-modified endograft (PMEG), Large fenestration, Stent strut relocation, Fenestrated endovascular aortic repair (FEVAR), Type I/IIIc endoleak

## Abstract

**Background:**

The Zenith® Fenestrated (ZFen) stent-graft is frequently configured with a strut-spanning large fenestration for superior mesenteric artery (SMA) incorporation. This has led some to relocate struts to create a strut-free fenestration and place a bridging stent. The aim of this study was to compare SMA outcomes with and without large fenestration strut relocation.

**Methods:**

We performed a retrospective review of a prospective database of patients undergoing fenestrated endovascular repair with ZFen between 2013 and 2019. Those with SMA incorporation using large fenestrations were included and separated into strut relocation (SR) and no relocation (NR) groups. Endpoints included procedural metrics, technical success, major adverse events, and target-vessel instability.

**Results:**

A total of 121 patients (77% male; mean age 76.1 ± 7.1 years) met inclusion criteria, including 94 with SR (78%) and 27 with NR (22%). A total of 369 target-vessels were incorporated, with a mean of 3.0 ± 0.2 per patient, and no differences between groups. Mean operative time, contrast volume, estimated blood loss, fluoroscopy time and radiation dose were lower (*p* < 0.001) with SR, attributed to increased experience with time. Overall technical success (SR: 100%, NR: 96%, *p* = 0.22) was 99%. At a mean follow-up of 32 months, there were two endovascular interventions for mesenteric ischemia. One resulted in SMA dissection requiring bypass in the NR group, the other was successful ballooning of the bridging stent with symptom resolution in the SR group.

**Conclusions:**

Relocating the spanning struts does not negatively impact procedural metrics or midterm outcomes. It may facilitate future endovascular interventions.

## Background

Advances in endovascular therapy and development of fenestrated endovascular aortic repair (FEVAR), has allowed for treatment of more complex aortic aneurysms (Oderich et al. 2017a; Resch 2013; Chuter 2007). Device designs have progressed from physician-modified endografts (PMEG) to custom manufactured (CMD) and off-the-shelf options. The Cook Zenith Fenestrated AAA Endovascular® (ZFen) (Cook Medical Inc., Bloomington, Ind.) was approved for commercial use in the United States (US) for aortic neck lengths between 4 and 15 mm. The fenestrated component is patient-specific, and designs may include up to three fenestrations, two of which can be the same type (Oderich et al. 2014a). Renal arteries are incorporated with nitinol reinforced, strut-free small fenestrations while the superior mesenteric artery (SMA) is often incorporated with a scallop or large fenestration (non-reinforced, often have crossing stent struts) (Oderich et al. 2014b).

As experience with the ZFen device has been gained, modifications to implantation have followed, including transition from using bare-metal stents to covered stents for small fenestration bridging (Vemuri et al. 2014). Bridging stents are not recommended for large fenestrations (Oderich et al. 2014b). However, the fear of device misalignment during implantation, the possibility of SMA “shuttering” and the potential difficulty associated with offering endovascular therapy for SMA disease post repair with ZFen has led some surgeons to relocate crossing struts of the large fenestration to the edge, allowing for placement of a bridging stent. Outcomes of this technique have not been reported. The aim of this study was to compare SMA outcomes with and without large fenestration stent relocation.

## Methods

All patients provided consent to be including in a minimal risk research protocol approved by institutional review board. We performed a retrospective review of a prospectively maintained database of patients undergoing FEVAR for TAAAs and PRAs between January 2013 and July 2019. We included only patients who underwent repair with ZFen utilizing a large fenestration for SMA incorporation. Patients were separated and analyzed based on whether large fenestration stent struts were relocated (SR) or not relocated (NR).

Demographic data and clinical characteristics were recorded. Procedural metrics were also collected for comparison between groups, including total operative time, fluoroscopy time, radiation dose and contrast volume. SMA outcomes included type Ic/IIIc endoleak and stent kink/compression (for SR group only), as well as occlusion and reintervention. Major adverse events (MAE) and 30-day mortality were also recorded.

### Stent strut relocation

Our technical approach to implantation of Zfen has been previously described (Manunga et al. 2018). The practice of large fenestration strut relocation was not adopted until after our 27st ZFen implantation with this configuration, in January 2015. This patient had a flow-limiting dissection at the origin of the SMA, and the spanning-strut with chronic ostial calcification were prohibitive in placing a stent, necessitating a bypass. After this date, all devices containing large fenestration underwent minor modifications while preserving device integrity.

**Fig. 1:**
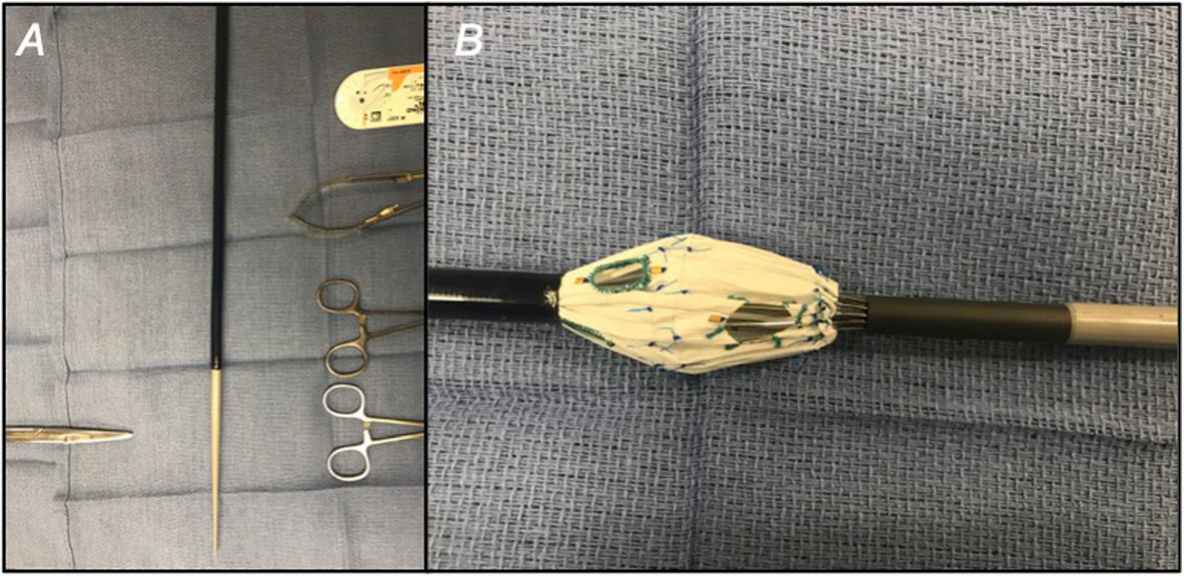
The Zenith Fenestrated AAA endoprosthesis is partially unsheathed, exposing the large fenestration intended for superior mesenteric artery incorporation

**Fig. 2:**
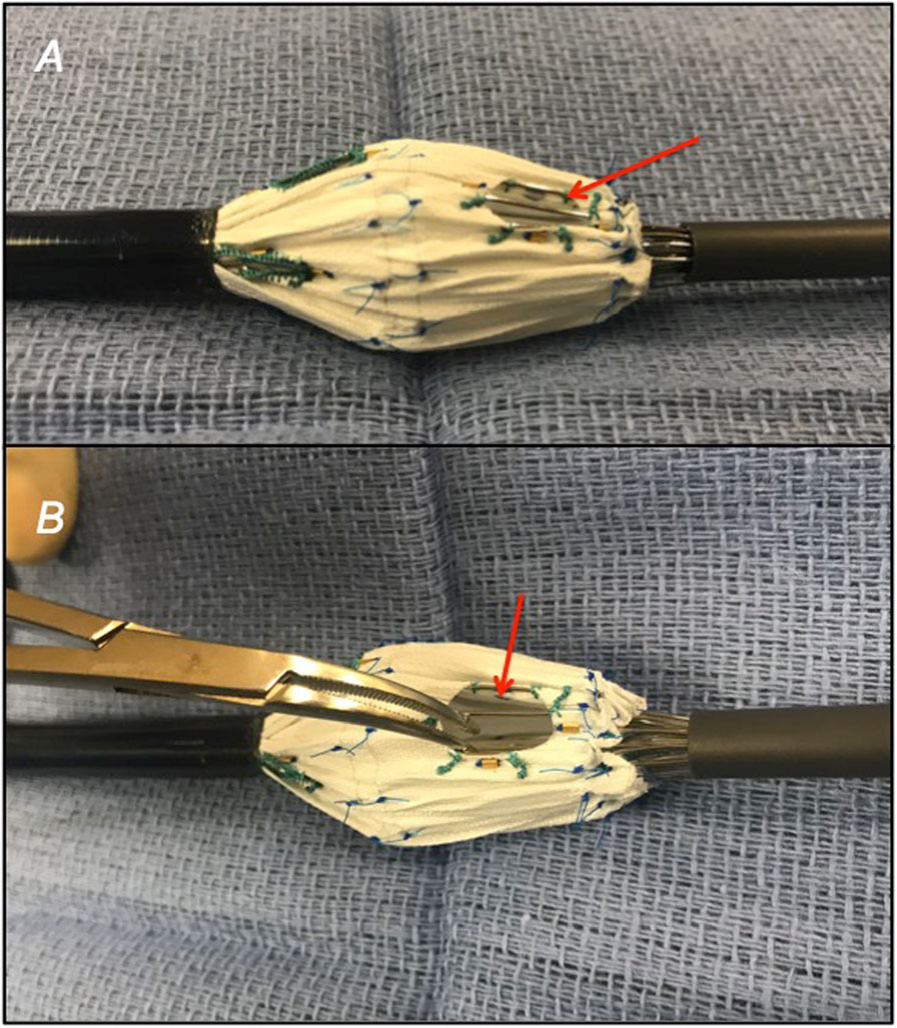
There is frequently a stent strut spanning the fenestration

**Fig. 3:**
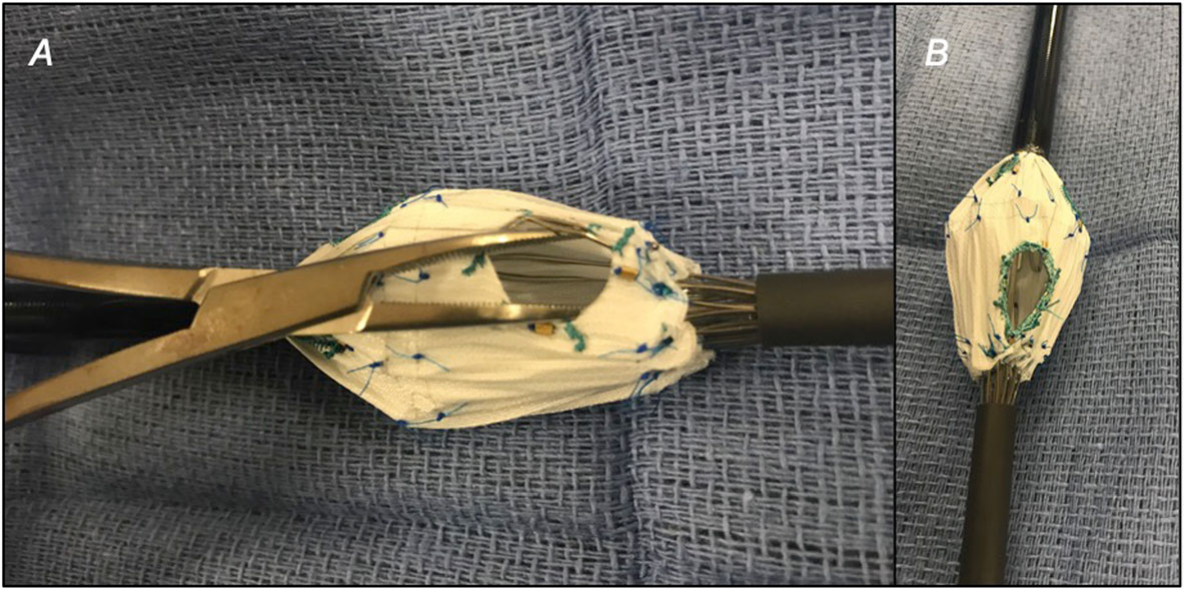
This is gently relocated to the edge of the fenestrationg with hemostat clamps (**a**), and sutured in place with a locking Ethibond suture that is sewn around the entire fenestration for reinforcement (**b**)

**Fig. 4:**
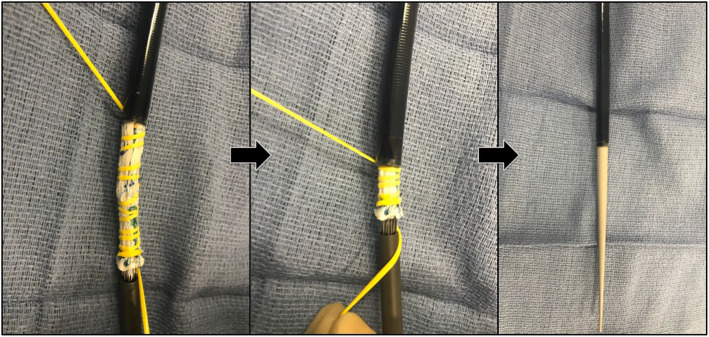
The device is then reconstrained with a silastic vessel loop to facilitate resheathing

The process consists of partially deploying the fenestrated device on a sterile back table until the large fenestration is exposed (Fig. 1). Crossing struts are gently bent using a hemostat and relocated from the middle (Fig. 2) to the fenestration edge (Fig. 3a). A 5–0 running Ethibond suture is used to secure the entire strut to the periphery of the fenestration (Fig. 3b). Suturing the edge of the fenestration further provides reinforcement to the fenestration and prevents fabric tear during flaring of the proximal bridging stent graft. Additionally, a running versus interrupted suture configuration is utilized to prevent any potential space for inadvertent catherization between stent strut and graft fabric during target vessel stenting. A silastic vessel loop is used to reconstrain the stent graft to allow resheathing (Fig. 4). All target vessels, including the SMA are bridged with iCAST® balloon-expandable covered stents (Atrium Maquet, Hudson NH).

Primary endpoints were procedural metrics and mid-term SMA outcomes. Secondary outcomes were 30-day mortality and MAE (acute kidney injury, estimated blood loss (EBL) > 1 L, myocardial infarction (MI), stroke, respiratory failure, spinal cord ischemia (SCI) grade 3a-3c, bowel ischemia requiring intervention). Technical success was defined as successful incorporation of all intended target vessels, including the SMA with either NR or SR techniques without conversion to an open repair.

### Statistical analysis

Results of continuous variables are reported as mean ± standard deviation (SD) or as median (interquartile range) depending on data distribution. Categorical variables are reported as frequencies and percentages. Differences in continuous variables were assessed using student’s t-test if normally distributed and the Wilcoxon rank-sum test if skewed. The analysis of discrete variables was performed using the chi-squared test or Fisher’s exact test where appropriate. Statistical significance was defined as a *P* < 0.05. All analyses were performed using Stata version 15.1 (College Station, TX).

## Results

### Patient characteristics

A total of 216 patients were treated using FEVAR during the study period, including 121 patients (56%) with ZFen device using large fenestrations, who were included in the study. There were 94 patients (78%) with SR and 27 patients (22%) with NR. The majority (77%) were males with a mean age of 76.1 ± 7.1 years. The most common patient comorbidities were hypertension in 119 patients (99%), coronary artery disease (CAD) in 113 patients (94%), and tobacco use in 112 patients (93%). More patients had chronic obstructive pulmonary disorder (COPD) in the NR group (67% versus 41%, *P* = 0.018) (Table 1). There were 63 patients with PRA (52%), 52 with juxtarenal aortic aneurysms (43%), and 6 with TAAA (5%).

**Table 1:** Clinical characteristics of 121 patients undergoing fenestrated endovascular aortic repair (FEVAR) using the Zenith Fenestrated AAA Endovascular® with large fenestrations for superior mesenteric artery incorporation

	**Total** **(*n*** **= 121)**	**No relocation** **(*n*** **= 27)**	**Relocation** **(*n*** **= 94)**	***P*** **value**
	*n (Percent) or Mean ± Standard Deviation*			
**Male**	92 (77)	22 (81)	70 (75)	0.50
**Age**	76.1 ± 7.1	76.0 ± 8.7	76.1 ± 6.6	0.95
**Hypertension**	119 (99)	27 (100)	92 (99)	1.00
**CAD**	113 (94)	25 (93)	88 (95)	0.65
**Smoking**	112 (93)	24 (89)	88 (95)	0.38
**Hyperlipidemia**	110 (92)	27 (100)	83 (89)	0.11
**COPD**	56 (47)	18 (67)	38 (41)	0.018
**CKD III-V**	49 (41)	11 (41)	38 (41)	0.99
**Diabetes**	14 (12)	2 (7)	12 (13)	0.73

*CAD* Coronary artery disease, *CKD* Chronic kidney disease, *COPD* Chronic obstruction pulmonary disease

### Procedural metrics

A total of 369 target-vessels were incorporated, with a mean of 3.0 ± 0.2 per patient and no differences between the two groups (*P* > 0.05). Technical success was identical between groups (SR -100%; NR -99%, *P* = 0.22) and there were no conversions to open repair. There was one patient in the NR group in whom the right renal artery was not successfully incorporated. There was perfusion of both renal arteries with no endoleak. The renal artery was successfully stented at a later date. The median total operative time [210 min. (163–250) NR vs. 80 (72–90) min. SR, *P* < 0.001], contrast volume [67 mL (60–90) NR vs. 47 mL (35–57) SR, *P* < .001], fluoroscopy time [80 min. (61–94) NR vs. 51 min. (45–61) SR, *P* < 0.001], and radiation dose [1609 mGy (1105–1982) NR vs. 1090 mGy (978–1312) SR, *P* < 0.001] were lower in the SR group (Table 2). This was likely due to the learning curve, as NR was only performed at the beginning of our experience.

**Table 2:** Procedural metrics of 121 patients undergoing fenestrated endovascular aortic repair (FEVAR) using the Zenith Fenestrated AAA Endovascular® with large fenestrations for superior mesenteric artery incorporation

	**Total** **(*n*** **= 121)**	**No relocation** **(*n*** **= 27)**	**Relocation** **(*n*** **= 94)**	***P*** **value**
	*Median (Q1, Q3)*			
**Total operative time (min)**	88 (77, 157)	210 (163, 250)	80 (72, 90)	< 0.001
**Fluoroscopy time (min)**	52 (46, 66)	80 (61, 94)	51 (45, 61)	< 0.001
**Radiation dose (mGy)**	1098 (989, 1670)	1609 (1105, 1982)	1090 (978, 1312)	< 0.001
**Contrast volume (mL)**	52 (37, 64)	67 (60, 90)	47 (35, 57)	< 0.001

*Min* Minutes, *mGy* milliGray, *mL* Milliliters

### SMA outcomes

Two patient required SMA reintervention for chronic mesenteric ischemia. One patient (3.7%) in NR group had a failed attempt at percutaneous angioplasty and stent placement due to dissection of the calcified origin of the SMA. While a wire and catheter could easily be advanced in to the true lumen, the presence of struts spanning the large fenestration made it impossible to accurately place a bridging stent. She ultimately underwent a right external iliac to the SMA bypass. One patient (1.1%) in the SR group had successful angioplasty of the prior iCAST stent from a brachial approach. There were no instances of SMA occlusion, stent kinking or compression. Furthermore, there were no type Ic or IIIc endoleak in either group (Table 3).

**Table 3:** Superior mesenteric artery (SMA) outcomes of 121 patients undergoing fenestrated endovascular aortic repair (FEVAR) using the Zenith Fenestrated AAA Endovascular® with large fenestrations for superior mesenteric artery incorporation

	**Total** **(*n*** **= 121)**	**No Relocation** **(*n*** **= 27)**	**Relocation** **(*n*** **= 94)**	***P*** **value**
	*Percent or Mean ± Standard Deviation*			
**Type I/IIIc endoleak**	0	NA	0	NA
**Kink/compression**	0	NA	0	NA
**Occlusion**	0	0	0	1
**Reintervention**	2 (2)	1 (4)	1 (1)	0.40

### Major adverse events

There were two deaths (1.7%) at 30-days, with one in each group (4% NR vs. 1% SR, *P* = 0.398). The rate of MAE was similar between the two groups, including new renal failure [2 NR (7%) vs. 2 SR (2%), *P* = 0.215], EBL > 1 L (0 NR vs. 0 SR), SCI 3a-c [1 NR (4%) vs. 0 SR, *P* = 0.223] and acute bowel ischemia [0 NR vs. 1 SR (1%), *P* = 1.00], with no instances of EBL > 1 L, MI, stroke, respiratory failure (Table 4). After a median follow-up of 32 (15–34, IQR) months, there were no aneurysm-related deaths or other patients with chronic mesenteric ischemia.

**Table 4:** Major adverse events (MAE) of 121 patients undergoing fenestrated endovascular aortic repair (FEVAR) using the Zenith Fenestrated AAA Endovascular® with large fenestrations for superior mesenteric artery incorporation

	**Total** **(*n*** **= 121)**	**No Relocation** **(*n*** **= 27)**	**Relocation** **(*n*** **= 94)**	***P*** **value**
	*Percent or Mean ± Standard Deviation*			
**30-Day Mortality**	2 (2)	1 (4)	1 (1)	0.398
**Any MAE**	5 (4)	3 (11)	2 (2)	0.073
**Renal Failure**	4 (3)	2 (7)	2 (2)	0.215
**SCI grade 3a-c**	1 (1)	1 (4)	0 (0)	0.223
**Bowel ischemia**	1 (1)	0 (0)	1 (1)	1.00
**EBL > 1 L**	0	0	0	NA
**Myocardial infarction**	0	0	0	NA
**Stroke**	0	0	0	NA
**Resp. failure**	0	0	0	NA

*MAE* Major adverse event, *SCI* Spinal cord ischemia, *EBL* Estimated blood loss, *Resp.* Respiratory

## Discussion

Misalignment of the SMA with ZFen and resultant complications have been a concern and previously investigated for both scallops and large fenestrations (Oderich et al. 2014b). There is some variation in practice and in the literature. Motta et al. evaluated outcomes of selective SMA stenting for scallops in 39 patients, with criteria for alignment stent use including concern for misalignment with SMA “balloon testing” and SMA stenosis. With strict criteria and rigorous follow up, there were no adverse events with selective SMA stenting at a mean follow-up of 22 months (Motta et al. 2019a). Ullery and colleagues examined the postoperative occurrence of “shuttering” or partial coverage of the SMA in unstented scallops. No patients presented with symptoms of mesenteric ischemia during the mean follow-up of 11 months. However, 50% of patients had some degree of SMA shuttering, ranging between 12% and 40%. The majority of patients had at least 21% shuttering, and while no acute or chronic mesenteric ischemia was noted, the mean follow-up period was only 11 months (Ullery et al. 2014). Conversely, in Lala’s series of 47 patients that compared the use of stent to no stent in large fenestrations and scallops, more MAEs were attributed to misalignment in the unstented group, with no misalignment occurring in the stented cohort. One patient developed SMA stenosis distal to the bridging stent that was remedied with angioplasty, similar to the case described in this report (Lala et al. 2016).

These studies only provide short-term data. There is a paucity of data looking at whether there is progression of partial SMA shuttering with scallops or fenestrations, and the long-term effects of partial shuttering on SMA patency and mesenteric ischemia. However, several observational studies have reported change in anatomy of target vessels after fenestrated repair. Kalder and colleagues demonstrated significant shift of target-vessel origin compared to the main device, independent of device and bridging stent used (Kalder et al. 2014). Furthermore, leaving arguably the most important visceral artery (SMA) partially covered by fabric is unsettling for most vascular surgeons.

The current ZFen configuration was conceived in 1999. Since then, significant knowledge has been gained and lessons have been learned thanks to results from physician-sponsored (PS) investigational device exemption (IDE) studies and industry-sponsored trials. Currently, none of the devices used in trials have struts spanning any target vessels. In fact, routine bridging of all target vessels with covered stents is now the norm rather than an exception. This is perhaps best evidenced by the Zenith p-Branch®, developed after the ZFen as an off-the-shelf option for juxtarenal aneurysms, with a strut free SMA fenestration for bridging stent placement (Kitagawa et al. 2013). This is also the case for most physician-modified fenestrated stent grafts (Tenorio et al. 2019a, b; Mirza et al. 2020; Oderich et al. 2017b; Motta et al. 2019b; Sveinsson et al. 2015). Therefore, we believe that performing a minor modification of ZFen device by displacing struts of the large fenestration to the side in order to bridge the SMA fenestration with a covered stent is prudent, logical and safe. This is particularly reasonable since doing so allows for an unobstructed flow to the SMA, eliminates the possibility of shuttering, makes re-intervention on target vessel easier, and does not compromise the long-term integrity of the device as demonstrated by our results. The issue of spanning struts across SMA fenestration has been eliminated in Europe, where subsequent device designs have incorporated the use of strut-free 8-mm fenestrations in additional to the traditional two small fenestrations utilized for the renal arteries. However, in cases where custom manufactured and off-the-shelf devices are not suitable, such as emergent presentations, PMEGs are a valuable option. In these cases, patient anatomy will often dictate that the SMA fenestration be made in a location where there is a crossing strut. This study demonstrates the safety and feasibility of strut relocaton, without detrimental effects.

While placement of bridging stents in visceral arteries ensures excellent perfusion, vessel catheterization carries an inherent risk of dissection, embolization, and small branch perforation that can sometimes be fatal. However, Motta et al. reported no increase in complications with placement of bridging stents in CA and SMA (Motta et al. 2019b). Starnes et al. described SMA incorporation with PMEGs using large fenestrations without routine use of SMA bridging stents (Starnes et al. 2016, 2017, 2018; Starnes 2012). On evaluation of their learning curve over an 8-year period with 136 cases there was one case of transient bowel ischemia that “resolved with medical management,” and another case of mesenteric ischemia, “unrelated to the implant or procedure,” that was treated with a celiac stent 4 years postoperatively (Starnes 2012). While the reasoning for stenting the celiac artery rather than the SMA in patient with mesenteric ischemia was not explained, one can infer that struts spanning the SMA fenestration made it difficult, if not impossible to stent the SMA (Starnes et al. 2017). This was certainly the case in our experience, where trying to stent an SMA that had struts spanning it resulted in a dissection of this critical vessel and conversion to an open bypass.

Starnes et al. also reported on the use of automated planning software to determine fenestration location, using algorithms that account for angulated aortic anatomy. The 97% rate of target-vessel incorporation demonstrates the importance of preoperative planning for FEVAR in accurate placement of fenestrations adjacent to the intended vessels (Starnes et al. 2018). However, we maintain our preference for SMA stenting due to uncontrollable variables such as tortuosity and angulation that can exert variable degrees of rotational force on the endograft and result in fenestration misalignment unless each target vessel is stented. This is partially exemplified by the reports on shuttering mentioned previously (Ullery et al. 2014). Additionally, while the rate of mesenteric ischemia is low with complex endovascular repair of TAAA and PRA (Oderich et al. 2017b), in our experience an SMA bridging stent facilitated endovascular rescue.

There were two (1.7%) deaths in our series, one in each arm. The first death occurred in a frail 91 year-old male in the NR group with a juxtarenal aortic aneurysm repaired using a three-fenestration device. Unfortunately, the patient developed postoperative paraplegia that improved after cerebrospinal fluid drainage and increased mean arterial pressures. Due to respiratory failure with ventilator dependence and the patient’s frail state, the family decided to withdraw care and she expired on post-operative day three. The second patient underwent successful exclusion of the aneurysm and stenting of all target vessels, including the SMA with excellent angiographic result but developed abdominal pain on postoperative day one with elevated lactic acid. On exploratory laparotomy, he was found to have diffuse and scattered ischemia of the small and large intestines. After multiple trips to the operating room for bowel resection, it was determined that short gut syndrome was likely and the family decided to withdraw care. In both cases, mortality was due to microembolization that likely occurred during attempts to align the device with fenestration. The first patient embolized to the lumbar and internal iliac arteries and the second patient embolized to the SMA and branches.

### Study limitations

The limitations of this study reside in the fact our group has a good experience with PMEG, having trained at a facility that routinely performed these procedures prior to obtaining an PS-IDE. For this reason, deploying the graft on the sterile back table, performing this minor modification and sheathing is very safe and does not take more than 15 min. This process, however, can be challenging for people with limited experience with back table device modifications. One needs to practice with demonstration devices, carefully examine fenestrations extracorporially under fluoroscopy, to ascertain there is no twisting or kinking before introducing the device in to the patient.

## Conclusions

Minor modifications of the large fenestration in the Zenith Fenestrated endoprosthesis, by relocating the spanning stent struts is safe and does not negatively impact procedural metrics, SMA outcomes, or major adverse events. To the contrary, it may facilitate future endovascular SMA interventions if needed.

## Data Availability

The datasets used and or analyzed during the current study are available from the corresponding author upon reasonable request.

## Abbreviations

FBEVAR: Fenestrated branched endovascular aortic repair
ZFen: Zenith Fenestrated Fenestrated AAA Endovascular®
SMA: Superior mesenteric artery
TAAA: Thoracoabdominal abdominal aortic aneuryms
PRA: Pararenal abdominal aortic aneurym
SR: Strut relocation
NR: No relocation
MAE: Major adverse events
SCI: Spinal cord ischemia
AKI: Acute kidney injury
EBL: Estimated blood loss
HTN: Hypertension
CAD: Coronary artery disease
COPD: Chronic obstructive pulmonary disease
MI: Myocardial infarction
PMEG: Physician modified endograft
